# Rohan Fernando: a road from Sri Lanka to Ames

**DOI:** 10.1186/s12711-022-00704-y

**Published:** 2022-02-04

**Authors:** Daniel Gianola, Rodolfo J. Cantet, Jack C. M. Dekkers, Miguel Pérez-Enciso

**Affiliations:** 1 University of Wisconsin-Madison, Madison, WI USA; 2 Universidad de Buenos Aires-INPA-CONICET, Buenos Aires, Argentina; 3 Iowa State University, Ames, IA USA; 4 ICREA, Universidad Autónoma de Barcelona, Barcelona, Spain

About one year ago, some of us learned that Rohan Fernando had announced his intention to retire from his position as Professor of Animal Science at Iowa State University. We felt that it would be appropriate to prepare a volume honoring his important contributions to quantitative genetics, especially to animal breeding. With that idea in mind, many of his collaborators and colleagues (past and present) were contacted and the idea was received with great enthusiasm. *Genetics Selection Evolution* (*GSE*) was considered to be very fitting as outlet and Jack Dekkers helped greatly to facilitate the task of publishing this collection of papers, prepared by current and past associates and students. The series touches many of the areas where Rohan's work was influential, such as estimation and prediction in populations undergoing selection, Bayesian methodology, theory and methods for a finite number of loci situations, genetic evaluation in crossbreeding populations, prediction with high-dimensional genomic data, statistical computing of large data sets, and software development. Each of the papers was subjected to the type of refereeing that is standard for *GSE*. Rohan is a coauthor of some of the papers, without realizing that they, ultimately, would appear in a collection recognizing his work!

Professor Fernando was born in Sri Lanka in a family with strong connections with agriculture, so he developed an interest in livestock. He attended Aquinas School in Colombo, a Catholic junior college, and then came to the United States, where he completed a Bachelor of Science in Animal Science at California State University in Fresno. He carried out postgraduate study at the University of Illinois at Urbana-Champaign, where he obtained Masters and Ph.D. degrees, with thesis work focusing on methodology in populations undergoing assortative mating and selection. He then continued his career as a tenured faculty member, first at the University of Illinois at Urbana-Champaign (1985–1996) and as Professor at Iowa State University thereafter.

He has been a productive scholar, with close to 15,000 citations and an h-index of 57 in Google Scholar; excellent marks in a small field. His six most cited papers include his seminal work on best linear unbiased prediction for marker assisted selection, his pioneering of Bayesian methods in animal breeding (at a time when Bayesianism was considered heretical), clarification of the role of markers in Bayesian regression models, including highlighting the importance of genetic similarity in prediction, and development of new methods for Bayesian variable selection. He also was a coauthor of the first paper pointing out the potential contribution of kernel methods to genome-enabled prediction.

In addition to many contributions in the areas mentioned above, Rohan's deep insights into difficult problems, coupled with his ability to arrive at elegant and convincing solutions, made him an ideal person to collaborate with, and to engage in discussion. During his career, he has assisted hundreds of students and collaborators, in a passionate and firm, albeit generous, quiet and humble, style. Those of us who have had the privilege of counting Rohan as a friend know that behind his sparse rhetoric, there is a person with very high ethical and spiritual dimensions. He has been extremely helpful to many, representing a sort of "Sister Theresa of Calcutta-equivalent" of animal breeding; always giving, seldom requesting.

Apart from being an avid reader of biblical material, he has always enjoyed company with his friends and family, good food and wine, and traveling, and he has mastered several “foreign languages”, such as C +  + , Matvec, and more recently Julia. With a nice smile, he could tell a friend "Go to hell" (he never did it, though), and make the recipient of the statement quite happy. We wish him luck in his next assignment where he will enjoy life and love in a land of oranges, wine, and electric cars, California, but beware of the earthquakes!

Since he has been, in some sense, a "best seller" in our field, below are excerpts from statements made by persons whose life was influenced in one way or another by Rohan. The order of their comments is approximately chronological, trying to match various stages of his development as a scientist. After reading these remarks, we encourage you to study the papers that are included in this series through which Rohan's influence on the ideas presented in the collection will become evident.

## Daniel Gianola (University of Wisconsin, United States)

My first two guinea pigs (also known as graduate students) at the University of Illinois at Urbana-Champaign (1978) were Wilson Nelson Mulenga Mwenya and Rohan Luigi Fernando. Both were excellent but different from each other in interests. Mwenya became a Dean at the University of Zambia, whereas Rohan chose scholarship. At that time, I had developed a "monster course" that covered essentially everything that was not being taught elsewhere, and with an important coverage of BLUP (best linear unbiased prediction) a very hot, mandatory, and mystical topic at that time. Most of us could follow Henderson's algebra, but without understanding the implications. Mwenya and Rohan took the course and both did well. A problem, however, was that Rohan would not ask questions in class (typical of him) but would later show up in my office with questions for which I did not have a readily available answer. To get rid of this "nuisance parameter", I would give him arcane books on mathematics and linear algebra and command: "read this and you will understand". That did not work either, because he would return with questions about the books, so my problems got worse instead of better! Eventually, we helped each other understand important foundational issues from our field, some of which we continue to discuss. Apart of the mentor–mentee relationship, we later became colleagues and friends.

At that time, graduate students in animal breeding were beginning to take classes in mathematical statistics, and we gradually evolved from writing linear models into thinking in terms of joint, conditional, and marginal distributions. When Sotan Im and Jean-Louis Foulley (INRA, France) came to Illinois for a sabbatical, both being powerful mathematicians, our mental entropy about statistical theory and its relationship to quantitative genetics gradually began to dissipate. I believe their contribution was crucial in Rohan's development as a quantitative scientist, and we finally understood the limitations and shortcomings of Henderson's paper on BLUP under selection. It took us a few years of discussion, even with Henderson, who was a recurrent visiting professor at the University of Illinois, to get to that point.

When I returned from a sabbatical in Jouy-en-Josas in 1982, I was fully convinced that the Bayesian approach would provide a solution to almost any problem in quantitative genetics. In fact, it has been used even to evaluate the statistical plausibility of the existence of a deity (not surprisingly, the prior is heavily influential). I narrated my Bayesian epiphany to Rohan, an experience that was equivalent to defending an innocent person in a trial organized by the Spanish Inquisition or the Ku-Klux-Klan. We found common ground and a paper emerged on “Bayesian stuff for animal breeders”. Without my interactions with Rohan, and with Daniel Sorensen, I would have never understood Bayes in a proper manner.

I have known Rohan for more than four decades and he has been an extremely important influence in my life as a scholar. I could narrate many anecdotes, but space is limited. However, I would like to end the statement with the following sentence: "If you were a coach, he should be in your starting five. If you do not love the man, go and see a shrink: you may be ill".

## Katherine Hanford and Stephen Kachman (University of Nebraska-Lincoln, United States)

Rohan has influenced our lives in many ways, both personally and professionally. We have been friends and colleagues of Rohan since we met as graduate students in Daniel Gianola's lab at the University of Illinois at Urbana-Champaign in 1981 and, over the past four decades, Steve and Rohan have continued their collaboration. We have numerous photos of Steve and Rohan sharing a laptop or writing on a white board or a sheet of paper, working on problems ranging from how to implement a statistical method in software, deciphering a bit of statistical theory, incorporating genomic information into genetic evaluations, and the pros and cons of Rohan's latest favorite programming language. Whether spending time at Iowa State University, serving together on various committees and projects, or getting together at meetings, we always looked forward to the opportunity to spend time with Rohan, where Steve could be assured to leave with a new idea to pursue and Kathy would be caught up on Rohan's kids.

## Rodolfo J. C. Cantet (Universidad de Buenos Aires-INPA-CONICET, Argentina)

Dr. Fernando and I met at the University of Illinois at Urbana-Champaign back in 1986, and he acted as my thesis codirector until I finished my PhD in 1990. I have many fond memories of those days. With Rohan, I learned to address hard research problems in quantitative genetics and genetic evaluation without much fear. Rohan, with his positive and generous contribution, helped me to learn how to find a solution. Actually, I believe he induced in his students the perception that we were the ones who had solved the problem, when in fact it was him who had led us to that point.

A paragraph in Wikipedia's description of the contributions of Dr. Fernando refers to the covariance between relatives for marked quantitative trait loci (QTL) and for multi-breed populations. I remember telling Rohan in 1988 about composite breeding and the need to develop a theory for the covariance between relatives in admixed breeds. A conversation of half an hour was enough to set Rohan in motion. He solved the problem for additive and additive plus dominance models and proposed an implementation of the additive model for evaluation of a two-breed composite.

I was impressed by his 1989 BLUP and marked QTL paper with Mike Grossman. In that publication Rohan derived the covariance between additive effects conditional on identity-by-descent (IBD) using his own and thorough reasoning. He showed how probabilities of IBD are functions of the recombination rate between a causal variant and the marker gene. This derivation is remarkable and emphasizes an individual mode of transmission of the genome that is affected by the pedigree. The work of Professor Fernando on IBD and covariance matrices of genetic effects has been extensive.

These statements reflect my view of what Rohan's contributions were and of what Rohan did for my thinking and education as a researcher in quantitative genetics. Animal breeders should all be grateful to him for his insight and intellectual generosity.

## Joel I. Weller (Agricultural Research Organization, The Volcani Institute, Israel)

I met Rohan when I did my first sabbatical at the University of Illinois at Urbana-Champaign in 1987–1988. We are nearly the same age but could not have more different early biographies, even though we both have strong religious beliefs. Daniel Gianola called Rohan a “Biblical Bayesian”, and that is probably a pretty-good description. I have therefore decided to include the following abstract of a thesis (Leah Dodell, "Revisiting Biblical Games in a Bayesian Framework", Ph.D., 2013, Emory University) in my congratulations on Rohan's retirement.

"In this paper, I revisit a few of the most debated tales from the Old Testament and model them in the framework of Bayesian games. I model three situations - Jacob's deception of Isaac, G-d's ten plagues, and Abraham's sacrifice - as dynamic games with private information. By solving for the Perfect Bayesian Equilibria that occur in the Torah, I find conditions that must hold for characters to be willing to take the actions that they do. I also examine how characters' actions would have changed if they had held different values. My results shed light on which interpretations of biblical stories hold the most weight when characters maintain consistent beliefs and act upon them in a sequentially rational manner."

From someone who retired three years ago, I can say that I highly recommend it. Best of luck in whatever you plan to do, but do not forget to study the Bible.

## Michael Lynch (Arizona State University, United States)

One of the greatest influences of my entire career came in the early 1980s at the University of Illinois at Urbana-Champaign, when I struck up a relationship with what was then one of the strongest groups of theoreticians in the world in quantitative genetics: Rohan Fernando, Jean-Louis Foulley, Daniel Gianola, Michael Grossman, and Charles Henderson. One of the most memorable sets of events involved a reading group focused on classical papers in the field, including Fisher 1918 and going even further back to Pearson's papers. Rohan and Dan set a frighteningly high bar by writing highly lucid 20-page sets of notes on each paper, which I still have today. Their presentations provided an extraordinarily clear and rigorous overview of some of the most difficult foundational papers in the field. I was able to bring a more evolutionary biology perspective, and jointly we put together an extraordinary couple of semesters with long sessions, that probably should have been recorded.

## Miguel Pérez-Enciso (ICREA, Universidad Autónoma de Barcelona, Spain)

Professor Fernando always smiles. Rohan is a rather humble person who shies away from the center of the stage and who does not discuss openly in public. Yet, he can spend hours calmly discussing with a colleague in private on a recondite issue he had encountered on a well-known and accepted procedure. His understanding of statistical issues in quantitative genetics is simply impressive.

Strangely for a highly self-contained scientist, up to this point, he has not authored a paper without coauthors. This may be a coincidence but it may also be a result of Rohan always looking for a partner to share knowledge with and to help.

His prose is terse and sober, as is his personality. I remember the first paper that we wrote together at the University of Illinois at Urbana-Champaign. I presented a first draft to Rohan, and we then spent an entire morning reviewing it word by word, removing as many as possible, and positioning each colon and semi colon as carefully as a surgeon would do with a scalpel in a delicate surgery. When I would talk to him while he was at Iowa State University, Professor Rohan was always excited by a new programming language, be it C +  +  or Julia, talking excitedly about how one could use it to program a famous differential equation as elegantly as possible.

Professor Fernando is author of over 200 publications with colleagues from all over the world, including the authors of this paper. They have covered, over the years, numerous topics that vary from Bayesian theory to plant breeding. They all share a clear objective of solving methodological problems, usually employing novel, surprising, and highly original solutions. Many of his works have had a profound influence on the field. Perhaps his most influential and premonitory work was "Marker assisted selection using best linear unbiased prediction", coauthored with Michael Grossman in 1989. In that work, he derives the relationship matrix and its inverse when using a marker and a QTL. The key contribution was that he treated the QTL allele effects as random, anticipating genomic selection principles by a decade. At a time when everybody was looking for candidate genes and excited about marker assisted selection, this work was amply cited but perhaps its philosophical implications were not fully understood at that time.

## Robert C. Elston (Case Western University, United States)

Rohan spent a postdoctoral year with me in New Orleans in 1991–1992. Chris Stricker was also with me at the same time and I believe this was when they first met; in any case, they soon realized they had a common interest and they became coauthors of at least seven papers. They had each come to me knowing that I was developing software to calculate likelihoods on large human pedigrees and they worked with me to develop new algorithms in this area. However, their real interest was in livestock pedigrees, which have many more inbreeding loops than are typically found in human pedigrees; and so they developed an algorithm that cut the numerous loops, but which gave a good approximation to the likelihood.

As with all good students – and Rohan was no exception – there comes a time when it is no longer clear who is the mentor and who the mentee. I hope I have been a good influence on Rohan; I know he has been a good influence on me.

## Max F. Rothschild (Iowa State University, United States)

It has been a great personal and professional pleasure to know and work with Dr. Rohan Fernando in the Animal Breeding and Genetics group at Iowa State University these many years. I first met Rohan when he was a graduate student with Dan Gianola at the University of Illinois at Urbana-Champaign. I always enjoyed his positive attitude and of course his interesting insights in solving statistical and genetics problems. While I was leader of the Animal Breeding and Genetics group at Iowa State University, I was instrumental in getting him and Jack Dekkers hired and these colleagues have worked well together and been great collaborators for the group, department, and around the world.

Rohan, as everyone knows, is an outstanding quantitative geneticist and also he has helped to not only develop theory but also program solutions for practical problems. His contributions in these areas have been significant.

People who also know Rohan know that, while extremely helpful and friendly, he does not seek out people, rather they come to him. In this regard, I have often joked that he sits in his office or “cave” and we need to go push him out or join him. I and a few of my students have collaborated with him on several occasions and his efforts greatly improved the research and work of my students. His is a patient mentor and works hard to explain even the most difficult concepts and has successfully lectured around the world.

Professor Rohan Fernando’s contributions to Animal Breeding and Genetics and statistical applications in our field have been considerable. It is my hope that he will continue to contribute not only his ideas to the field but continue his friendship with his many colleagues here in Iowa and around the world.

## Dorian Garrick (Massey University, New Zealand)

Rohan Fernando has always been quiet and unassuming, so his presence could easily go unnoticed. I first met Rohan Fernando in the mid 1980's and quickly learnt that his publications and his presentations were carefully thought out, from first principles, and always very thorough. I immediately had a lot of respect for his work, particularly his 1989 paper on “Marker-assisted selection using BLUP” when it first came out.

I did not really get to know Rohan well until I joined him at Iowa State University in 2007 and began working closely with him. His first big contribution to my academic career was in the understanding of Bayesian approaches to some animal breeding and related problems, particularly BayesA and BayesB. Most of my knowledge on linear model theory and applications was based on my pre-doctoral interactions with Dr. Robert Anderson and with Arthur Gilmour during his Ph.D. studies, and then during my Ph.D. at Cornell from Drs. Searle, Quaas, Van Vleck, Henderson, Pollak, and Coleman. I had been taught a fair bit about Bayesian inference by George Casella when I was a Ph.D. student at Cornell in the mid 1980's. But, at that time we did not know how to apply it to real world problems associated with estimation of variance components or genetic evaluation. Or at least I did not know how to do it.

Daniel Gianola and his students, particularly Alicia Carriquiry, made many nice presentations on Bayesian inference at conferences, promoting the appealing concepts, but they always stopped short of educating me as to how it could be done in practice. So, I never came away from those activities with any better idea of how to apply it in real life. My first landmark in understanding real-world applications of Bayesian inference was at a course that Martin Tanner held in Armidale. There, I learned about Markov Chain Monte Carlo, Metropolis–Hastings, Gibbs sampling, data augmentation, and other treasures, but mostly applied to problems quite unlike those involving large-sparse sets of mixed model equations like the ones we routinely used in REML estimation of variance components, or in genetic evaluation.

My next landmark was in reading the 2001 Meuwissen, Hayes, and Goddard paper. I did not spend as much time coming to grips with their BayesA and BayesB methods as I should have, but their so-called BLUP approach was as familiar to me as bread and butter. BayesA and BayesB would have had a much bigger impact on me at the time if we had real-life SNP chip data, but at that time I was still working with microsatellite markers.

Soon after my arrival at Iowa State University, I received my first 50 k bovine genotype data on some 1000 animals and I started working with Rohan on how we might make inference. Rohan convinced me of the merit of BayesA and BayesB and helped me understand some of the intricacies of those algorithms. The light-bulb finally turned on when Rohan again patiently demonstrated to me the issues of Gibbs sampling and I realized the very close similarity between single-site Gibbs sampling and Gauss–Seidel iteration, then the close similarity between joint Gibbs sampling and block Gauss–Seidel. The Gibbs sampler is a most amazing algorithm. Once I finally understood more about BayesA and BayesB, we could start thinking about what we might do to further improve the algorithms and to expand the so-called Bayesian alphabet.

Rohan moved his office next to mine when Dr. Richard Willham retired, so we would not have to pace the length of the corridor between his old office and mine. A typical scenario would involve me thinking up a hare-brained idea for improvement of an approach and suggesting it to Rohan. Sometimes he would immediately and politely point out its inadequacies. On a few occasions, when it warranted further consideration, he would go back to his pen and paper, or his whiteboard, and work the issue through. Then, often in the middle of the night, Rohan would start playing with small examples. That became a lot easier after 2012 when we started using Julia rather than R to prototype new ideas before C +  +  implementation. Rohan was one of the first to use Julia and has been responsible for much of its adoption by the animal breeding community. Communication became even easier for Rohan when Jupyter notebooks became available. Instead of emailing a snippet of R or Julia code, the norm became a Python notebook that contained the Latex markdown, the simulation, results, and usually graphs, making it much easier to share ideas with other interested parties.

Reminiscent of my memories of Dick Quaas, most of the activities Rohan has worked on have led to useful extensions of known concepts and much improved understanding. Those that appear in refereed journals represent only the tip of the iceberg of such endeavours. Rohan almost always reverts to the most basic principles when considering a problem. He never takes some of the results others claim for granted. When Rohan referees a novel paper, it means rederiving all the findings in the paper.

Rohan has made a remarkable contribution to the lives of many students, postdoctoral fellows, and young and old scientists. I consider myself very fortunate to have crossed paths with him, and to have been able to work closely with him on a number of problems over the last 20 years.

## Jack C. M. Dekkers (Iowa State University, United States)

One of the most brilliant yet modest scientists with whom I have ever had the chance to work with, along with one of the kindest and most patient persons that I have ever met. That is how I would describe Rohan.

I got to know Rohan after I started as a faculty member at Iowa State University in 1997. I had read some of his papers but, frankly, had trouble understanding them in full, mostly because I tend to approach problems from a more conceptual manner, in contrast to the mathematical and statistical rigor that Rohan employs. Yet, as we started to discuss problems in animal breeding, we were able to find much common ground, where our approaches complemented each other. Over two decades of fruitful and enjoyable collaborations followed, resulting in 56 coauthored papers, and still going strong.

I have always admired Rohan's rigorous and meticulous attention to detail when it comes to statistical and quantitative genetics, always striving to getting to the bottom of the problem and not taking anything for granted. I remember us visiting for hours in the late 1990's with Alicia Carriquiry's husband, Wolfgang Kliemann, who was a mathematics professor at Iowa State University. Wolfgang explained the mathematical basis of the Gibbs sampler and the Metropolis–Hastings sampler to us. And not until Rohan had fully convinced himself of their validity, was he willing to let Markov chain Monte Carlo replace, or rather, complement the likelihood-based philosophy that was ingrained into his approach to statistical genetics. Since that time, he has never looked back and made, and continues to make, tremendous and novel contributions to the field of Bayesian statistics applied to animal breeding and quantitative genetics. Even now, upon his retirement, he is embarking on the power of machine learning, getting to the bottom of it, convincing himself that it works, connecting it to his knowledge of likelihood and Bayesian methods, and employing it to solve problems in our field.

In addition to a being a superb scientist, Rohan has also been a great educator and mentor. The students, post-docs, and visiting scientists who have had the chance to work with him can attest to the close working relationship that he developed with each of them. Often, prior to Covid19, when you walked by his office, you would see a student or post-doc sitting next to him behind the computer, as they were working together on a program or a paper. Even now, during the pandemic, lengthy Zoom calls with students and post-docs have been the rule of the day for Rohan. The patience and collegiality that he has exhibited over the years with all graduate students and post-docs who he worked with, regardless of whether they were his own or not, is truly remarkable and enviable.

Rohan, my friend and colleague, enjoy your well-deserved retirement but, please, stay in touch.

